# Lesion search and recognition by thymine DNA glycosylase revealed by single molecule imaging

**DOI:** 10.1093/nar/gkv139

**Published:** 2015-02-24

**Authors:** Claudia N. Buechner, Atanu Maiti, Alexander C. Drohat, Ingrid Tessmer

**Affiliations:** 1 Rudolf Virchow Center for Experimental Biomedicine, University of Würzburg, Würzburg, Germany; 2 Department of Biochemistry and Molecular Biology and Greenebaum Cancer Center, University of Maryland School of Medicine, Baltimore, MD 21201, USA

## Abstract

The ability of DNA glycosylases to rapidly and efficiently detect lesions among a vast excess of nondamaged DNA bases is vitally important in base excision repair (BER). Here, we use single molecule imaging by atomic force microscopy (AFM) supported by a 2-aminopurine fluorescence base flipping assay to study damage search by human thymine DNA glycosylase (hTDG), which initiates BER of mutagenic and cytotoxic G:T and G:U mispairs in DNA. Our data reveal an equilibrium between two conformational states of hTDG–DNA complexes, assigned as search complex (SC) and interrogation complex (IC), both at target lesions and undamaged DNA sites. Notably, for both hTDG and a second glycosylase, hOGG1, which recognizes structurally different 8-oxoguanine lesions, the conformation of the DNA in the SC mirrors innate structural properties of their respective target sites. In the IC, the DNA is sharply bent, as seen in crystal structures of hTDG lesion recognition complexes, which likely supports the base flipping required for lesion identification. Our results support a potentially general concept of sculpting of glycosylases to their targets, allowing them to exploit the energetic cost of DNA bending for initial lesion sensing, coupled with continuous (extrahelical) base interrogation during lesion search by DNA glycosylases.

## INTRODUCTION

Base excision repair (BER) is the first-line defense to protect the genome from detrimental effects of cytotoxic and mutagenic DNA base oxidation, deamination and alkylation (1). Initial detection and removal of these DNA lesions within the huge excess of normal bases is achieved by DNA glycosylases. The question of how BER enzymes detect their target sites within the huge excess of undamaged bases remains largely unresolved. In general, DNA glycosylases have to compromise between the needs to minimize the time for lesion search and to maximize the selectivity for removal of their target base (2). Like other DNA binding proteins (3,4), under the *in vivo* conditions of high DNA concentration, DNA repair enzymes have developed the ability to bind nonspecific DNA with moderate affinity (5–7). One-dimensional diffusion in contact with undamaged DNA (sliding) and three-dimensional transfer among DNA segments (hopping) may then facilitate rapid lesion detection (4–6,8). Final target identification requires contacts with residues in the enzyme active site achieved by flipping of the damaged base into an extrahelical state. In the catalytic step, the base-sugar (N-glycosidic) bond of the everted base is cleaved, creating an abasic site in the DNA. Because these sites are highly susceptible to ssDNA breaks (9,10), abasic sites are protected by the glycosylase after base excision until further processing either by an apyrimidinic/apurinic endonuclease (for monofunctional glycosylases) or via an additional intrinsic apyrimidinic/apurinic lyase activity of the glycosylase (for bifunctional glycosylases). Finally, further downstream BER factors are recruited to complete the repair reaction and genomic integrity is regained (11).

In the lesion recognition complex, many DNA glycosylases [e.g. uracil DNA glycosylase (UDG), hOGG1 or AlkA] (11) have been shown to display a high density of polar phosphodiester interactions with the damaged strand and only a few contacts with the undamaged strand, leading to DNA backbone distortion (bending). The resulting protein-induced alternative DNA backbone conformations and induced torsional stress on the target nucleotide have been shown to lower the energetic barrier for base flipping (12–14). Hereby, the lower stability of DNA at damaged compared to homoduplex sites (15,16) facilitates DNA bending and base flipping at target sites (17,18) and enhances the probability for spontaneous base pair opening (base pair breathing). Currently, these processes are less well understood for nonspecific DNA and the question whether DNA glycosylases bend and flip undamaged DNA during lesion search is still a matter of considerable debate (19,20). The mechanisms underlying enzymatic base flipping have been studied for several glycosylases (e.g. UDG, T4-Pdg, hOGG1, AlkA) with different techniques (2,17,21–24). A major question is whether the proteins fulfill an active (inducing base flipping) or passive (stabilizing the flipped state) role in nucleotide flipping. For example, for UNG glycosylase, capture of transiently emerging bases (passive flipping) has been demonstrated at nontarget sites (24–27). On the other hand, the glycosylase hOGG1 and its bacterial homolog MutM were reported to exploit an active intrahelical base interrogation and extrusion mechanism (13,28). The particular (active or passive) mechanism of base eversion may hence vary for different glycosylases. Indeed, it seems likely that not only base extrusion but the entire process of target site search is optimized for the individual energetic requirements of the particular target sites of a specific glycosylase.

Focusing on the specific example of the human thymine DNA glycosylase (hTDG), we address the question of DNA lesion search strategies of DNA glycosylases by atomic force microscopy (AFM) imaging. hTDG belongs to the UDG protein superfamily (29) and is involved in BER as well as epigenetic gene regulation being responsible for active DNA demethylation (30–32). It shows a strong repair activity for uracil from G:U mispairs as well as for oxidized forms of methylated cytosine created by Tet enzymes, including 5-formylcytosine and 5-carboxylcytosine (5fC, 5caC) (33,34). In addition, hTDG maintains the integrity of CpG sites by selective removal of thymine from G:T mismatches caused by deamination of 5-methylcytosine (5mC). Notably, hTDG efficiently avoids futile repair of undamaged DNA; the enzyme excises thymine, a *normal* base, with 18 000-fold greater activity from G:T mispairs than from A:T pairs (35). Recent crystal structures of the hTDG catalytic core domain (hTDG_cat_) showed the enzyme in the lesion recognition state with the noncleavable substrate analog 2′-deoxy-2′-flouroarabinouridine (U^F^) or 5caC, and the product complex with the deamination product 5-hydroxymethyl uracil (5hmU) or an abasic sugar flipped into its active site (31,36–38). Interestingly, in three of these structures (31,36,38), the enzyme crystallized in a 2:1 complex with DNA, with one subunit bound to the target lesion (specific complex) and one subunit bound to undamaged DNA in close vicinity (nonspecific complex). Biochemical assays and kinetic studies confirmed dimerization (of the catalytic domain as well as the full length protein) under conditions of high and saturating [hTDG], but showed that a monomer of hTDG was fully capable of DNA lesion recognition and base excision (38,39). Due to the largely unstructured N-terminal domain, there are no crystal structures of full-length hTDG available. However, the structures of hTDG_cat_ bound to either abasic DNA, 5hmU, G:U^F^ or 5caC revealed several residues essential for nucleotide flipping (e.g. Arg^275^) and catalysis (e.g. Asn^140^) in the specific lesion recognition complex (31,36–38). In contrast, the dynamics of base extrusion are less well understood, in particular for undamaged bases, and it remains elusive whether hTDG flips DNA bases in an active or passive manner.

In our AFM studies, we use long (500–2000 bp) fragments of duplex DNA that is either nondamaged (homoduplex DNA) or contains a specific lesion at a well-defined position. To capture the enzyme in its lesion recognition state, we use the catalytically inactive hTDG variant N140A, which exhibits similar DNA binding affinity as the wild-type (wt) enzyme, but only very minor, residual base excision activity for G:U and no detectable activity for G:T mispairs (40,41). This enables us to study individual hTDG–DNA complexes during lesion search and detection in the absence of base excision. Our AFM data analysis of protein-induced DNA bend angles revealed that hTDG adopts two clearly distinguishable complex conformations both at damaged and undamaged DNA sites. Using mutational studies and a fluorescence-based nucleotide flipping assay, we confirmed an important role of the strictly conserved residue Arg^275^ in base flipping by TDG. Importantly, our data strongly suggest a lesion search mechanism of TDG that involves an initial damage-sensing step, which exploits mechanical properties of its target sites in DNA, coupled with (extrahelical) base interrogation. We present a model that summarizes our findings in the context of general implications for DNA lesion search by DNA glycosylases.

## MATERIALS AND METHODS

### Enzymes

hTDG wt and variants, N140A and R275A (all full length), were expressed and purified as described (40). All experiments with the native TDG targets G:T and G:U were carried out with the strongly incision impaired TDG-N140A variant (40), while wt or R275A variants [with residual base excision activity (40)] were incubated with the G:U analog G:U^F^ that cannot be processed by hTDG. Recombinant human 8-oxoguanine glycosylase (hOGG1) was obtained from MyBioSource as N-terminally his-tagged and purified protein with a concentration of 0.5 mg/ml as determined by Bradford assay.

### DNA substrate preparation

Linear specific DNA substrates for AFM (549 bp) were produced similarly as recently described (42,43). Briefly, DNA substrate preparation is based on the plasmid pUC19N (2729 base pairs, a gift from Samuel Wilson's laboratory, NIEHS, USA), which contains two restriction sites of the nickase Nt.BstNBI (New England Biolabs, NEB) in close vicinity. After nicking with Nt.BstNBI, the short ssDNA fragment between the nick sites (48 nt, Table 1, B) was removed by repeated heating and spin-filtering in the presence of an excess of the complementary oligonucleotide. The ssDNA gap was subsequently replaced by a target site containing DNA insert and the nicks between the original DNA and the insert were sealed by overnight incubation with T4 DNA ligase (NEB) at ambient temperature. The sequences of the inserts used as target site specific DNA substrates for hTDG and hOGG1 are listed in Table 1. Correct insertion was controlled by restriction enzyme assays using agarose gel electrophoresis after each step of the sample preparation as previously described (42,43) using BglII (NEB). Target site containing plasmids were then digested with the restriction enzymes NdeI and BspQI (NEB) yielding the specific, linear 549 bp DNA fragments for AFM imaging experiments. To investigate target site search processes, nonspecific DNA fragments (DNA substrates not containing a glycosylase target site) were further generated by digestion of undamaged pUC19N with NdeI and BspQI. To probe for an effect of DNA sequence context and/or DNA fragment length, a second nonspecific DNA substrate was further prepared with a length of 1813 bp, by digestion of undamaged pUC19N with BspQI and SspI. After restriction enzyme digestion, all DNA substrates (specific and nonspecific) for AFM imaging were purified by extraction of the corresponding band from agarose gel electrophoresis using the NucleoSpin Extract II Kit (Macherey-Nagel, Duren, Germany). The final substrates contain either homoduplex DNA (original DNA sequence as in Table 1, B) or a hTDG or hOGG1 target site at 46% of the DNA fragment length; a G:U (C, Integrated DNA Technologies), G:U^F^ (D, Midland Certified Reagents) or G:T mismatch (E, Biomers) in a CpG context or an 8oxoG:C base lesion (F, Midland Certified Reagents) as hTDG or hOGG1 target sites, respectively.

**Table 1.** DNA substrates

	DNA substrate	DNA sequence
A	Bottom strand	GGT CGA CTC TAG AGG ATC AGA TCT GGT ACC TCT AGA CTC GAG GCA TGC
B	Original Top	GCA TGC CTC GAG TCT AGA GGT ACC AGA TCT GAT CCT CTA GAG TCG ACC
C	G:U mismatch	GCA TGC CT(dU) GAG TCT AGA GGT ACC AGA TCT GAT CCT CTA GAG TCG ACC
D	G:U^F^ mismatch	GCA TGC CT(dU^F^) GAG TCT AGA GGT ACC AGA TCT GAT CCT CTA GAG TCG ACC
E	G:T mismatch	GCA TGC CT(T) GAG TCT AGA GGT ACC AGA TCT GAT CCT CTA GAG TCG ACC
F	8oxoG:C	GCA TGC CTC (8-oxo-G)AG TCT AGA GGT ACC AGA TCT GAT CCT CTA GAG TCG ACC
G	2-AP G:U^F^	ATG CAT GCC (2-AP)(dU^F^)G AGT CTA GAG G
H	2-AP nonspecific	AT GCA TGC C (2-AP)CG AGT CTA GAG G
I	2-AP 8oxoG:C	AT G CA T GC C (2-AP)(8oxoG)G AGT CTA GAG G
J	2-AP Counter 1	CCT CTA GAC TCG TGG CAT GCAT
K	2-AP Counter 2	CCT CTA GAC TCC TGG CAT GCA T

(A–F) DNA substrates for AFM experiments: only the 48 nt insert (C)–(F) is shown (see Materials and Methods). (B) shows the original sequence replaced by (C–F). (G–K) DNA substrates for AP-2 fluorescence assays. Oligonucleotides G and H were annealed with J; I was annealed with K. The bases that form a DNA mismatch or target site are indicated in brackets.

Specific DNA oligonucleotides for fluorescence-based base flipping assays were ordered with 2-aminopurine (2-AP) modification neighboring the target sites U^F^ or 8oxoG (Table 1, G or I, respectively, both from Midland Certified Reagents). For nonspecific DNA substrates, 2-AP was placed between two cytosines (H, obtained from Integrated DNA Technologies). Complementary oligonucleotides (J and K) were purchased by Sigma Aldrich. For annealing, oligonucleotides were mixed at equimolar ratio; oligonucleotides G (specific substrate) or H (nonspecific substrate) with J for hTDG studies and oligonucleotide I with K (specific substrate) and H with J (nonspecific substrate) for experiments on hOGG1.

### AFM imaging

Prior to AFM experiments, all DNA substrates were heated to 65°C for 10 min and slowly cooled down to room temperature to remove possible salt microcrystals formed on DNA upon storage. For analysis of hTDG interactions with lesion-specific DNA substrates, 50 nM of hTDG variants (N140A or R275A) were incubated with 2.5 nM target site specific (G:U, G:U^F^, or G:T mismatch in a CpG context) or nonspecific 549 bp DNA substrates. For experiments on hTDG with 1813 bp nonspecific DNA, 50 nM hTDG (wt or variant R275A) were incubated with 1.5 nM undamaged DNA fragments. All hTDG–DNA samples were incubated for 20 min at room temperature in AFM buffer (25 mM HEPES pH 7.5, 25 mM sodium acetate, 10 mM magnesium acetate). hOGG1 wt (150 nM) was incubated with undamaged DNA fragments (2 nM for 1813 bp DNA and 5 nM for 549 bp DNA) in AFM buffer for 10 min at room temperature. As a control, 25 nM protein (for both hTDG and hOGG1) were incubated as described above, but in the absence of DNA. Likewise, DNA substrates were incubated in the absence of protein at incubation concentrations of 2.5 nM for 549 bp DNA fragments and 1.5 nM for 1813 bp DNA fragments for determination of intrinsic DNA bend angles. For all samples, 20 μl of sample solution was deposited onto freshly cleaved mica (Grade V; SPI Supplies) after incubations, rinsed with ultrapure water, and dried in a gentle stream of nitrogen. All AFM images were collected in air with a molecular force probe 3D-Bio AFM (Asylum Research, Santa Barbara, CA, USA) in tapping mode using OMCL-AC240TS (Olympus) noncontact/tapping mode silicon probes with spring constants of ∼2 N/m and resonance frequencies of ∼75 kHz. AFM micrographs were captured at a scan speed of 2.5 μm/s with image sizes of 2 μm x 2 μm, 4 μm x 4 μm or 8 μm x 8 μm and pixel resolution of ∼2 nm.

### AFM data analysis

Utilizing Asylum Research software on Igor Pro, AFM images were plane-fitted and flattened to third order. Protein–DNA complexes were manually identified from the AFM images and analyzed in terms of binding positions on the DNA and DNA bend angle, excluding protein complexes on DNA aggregates and on DNA fragments that were cut off by the image margins. Statistical distributions of protein positions along the long DNA fragments can reveal preferences (specificities) for binding to a particular site within the substrates (e.g. here to a base mismatch within a CpG context located at 46% of the DNA fragment length). Substrate-binding specificities (*S*) of hTDG for its target site were quantified from Gaussian fits to position distributions of hTDG–DNA complexes as recently described (43,44). In short, complex positions were determined as the distance to the closer DNA fragment end normed to the full length of the DNA substrate by placing contours along the DNA backbone with Image J (NIH open-source software). We included only DNA fragments within two standard deviations (SD) from the center of a Gaussian fit to the DNA length distributions (165 ± 24 nm) in the analyses (from all experiments and a total of *n* = 1530 DNA strands). Position distributions were plotted as histograms using the software Origin Pro 8.5. Binding peaks of hTDG were observed at 0 and 46% in the position distributions, which were assigned as binding preferences for DNA fragment ends and for the position of the target lesion (located at 46% of DNA fragment length, see above, DNA substrate preparation), respectively. For quantitative analysis of binding specificities, we excluded DNA end-bound hTDG complexes from the histograms starting at 5% of DNA length and fixing the bin size (to 4%) for all individual experiments. Lesion specificity for a target site results in a peak at the specific site (∼46% DNA length), which can be fitted with a single Gaussian curve. Integration of the Gaussian peak area (*A*_spec_) provides the fraction of specific complexes. The fraction of nonspecific complexes is represented by the nonspecific area of the background (*A*_nsp_) and is calculated as the product of considered DNA length (45%) and the height of the background from the Gaussian fit (y_0_). With *N* = 494 binding sites (excluding 2x 5% of DNA fragment length), lesion specificity *S* is defined as (45):

(1) \begin{equation*} S = N*\frac{{A_{{\rm spec}} }}{{A_{{\rm nsp}} }} + 1 \end{equation*}

All Gaussian widths were fixed to the width of the Gaussian fit to the substrate with highest specificity (3.2%, for hTDG-R275A with G:U^F^) to directly compare binding specificities of hTDG variants for different DNA substrates. Specificities were calculated as average values from three individual experiments with SD derived from the variations between single experiments. Position distributions were produced from pooled data from three individual experiments.

Based on the protein position distributions, we separated specific hTDG–DNA complexes (bound at the target site ∼46% ± 2 SD) and nonspecific hTDG complexes bound elsewhere on DNA for further DNA bend angle analysis. DNA bend angles *β* are given as the deviation from a straight line through the DNA backbone:

(2) \begin{equation*} \beta = 180 - \alpha \end{equation*}

We determined *α* by placing two lines along the DNA backbone on each side of a protein complex by manual tangent overlay using Image J. All bend angle distributions in the presence of protein represent pooled data from three individual AFM experiments. Bend angle values for hTDG and hOGG1–DNA complexes were derived as the maxima of double or triple Gaussian fits to pooled bend angle distributions, with errors given by the half-width of the Gaussian (one SD). Pooled distributions were produced from three experiments. To determine percentages of different bend angle populations, we fixed all widths of Gaussian fits to the same value and integrated resulting Gaussian peak areas using Origin Pro. For the AFM studies with hTDG, we were able to focus on the catalytically inactive hTDG variant N140A in the main text as the mutant showed comparable DNA bending behavior as the wt [e.g. on nonspecific DNA bend angles of (31 ± 12)° (39%) and (65 ± 12)° (61%) for hTDG-N140A and (33 ± 11)° (46%) and (68 ± 11)° (54%) for hTDG wt, *P* = 0.478 using a one-tailed Student's *t*-test, Supplementary Figure S1]. Bend angle distributions for nonspecifically bound complexes were derived from experiments with DNA substrates that did not contain any lesion sites. However, analyses carried out on nonspecifically bound complexes on G:U or G:T mismatch containing substrates showed comparable distributions (data not shown). To avoid constraints on the measured DNA bend angles, only complexes with no neighboring protein on the same DNA or with distances of at least 50 nm to a further bound protein complex were included in bend angle analyses. AFM resolution limits do not enable separate distinction of two protein molecules bound within close distance (<∼30 bp or ∼10 nm) from each other on the DNA. To exclude the possibility of such closely bound molecules being mistaken for single complexes, we measured the volumes of the protein–DNA peaks in the images for incubations at low (25 nM) and high (2.5 μM) hTDG concentrations (Supplementary Figure S2). AFM volumes can be translated into approximate protein molecular weight (MW) via an empirically derived calibration curve (46). Our AFM system has previously been calibrated using a set of proteins with known MW (47), providing a linear relationship between protein MW and measured AFM volume (V): MW = (V + 5.9)/1.2. The resulting statistical volume distribution of DNA-bound hTDG indicated that the majority of complexes (≥80%) consists of only a single monomer of hTDG even at the high protein concentration of 2.5 μM (Supplementary Figure S2).

To determine intrinsic bending of specific DNA substrates (549 bp) in the absence of protein, we moved a mask comparable to the size of the protein to 46% of DNA fragment length corresponding to the position of the target site (see above, DNA substrate preparation) and measured DNA bending at this position as described above for protein-induced DNA bend angles. All individual intrinsic DNA bend angle distributions for the different DNA substrates in the absence of protein represent pooled data from two individual AFM experiments. We also measured intrinsic bending for 549 bp undamaged DNA substrates at this position (46% of fragment length) to exclude any influence of intrinsic DNA bending behavior due to sequence context at the position itself (Supplementary Figure S3A). Furthermore, we measured intrinsic DNA bending of nonspecific (undamaged) DNA fragments (1813 bp) (Supplementary Figure S3B) at regular intervals of 50 nm by moving a mask comparable to the size of the protein along DNA. The experiments were carried out in duplicate. As both controls for intrinsic DNA bending were very comparable [DNA bend angles of (0 ± 18) and (0 ± 23)° for 549 bp and 1813 bp substrates, respectively], we were able to pool these data to collectively represent nonspecific DNA bending.

### Fluorescence measurements

Nucleotide flipping activity of hTDG and hOGG1 was monitored using a 2-AP fluorescence-based base flipping assay. 2-AP is a fluorescent base analog of adenine and its fluorescence is highly quenched within a DNA duplex due to stacking interactions with neighboring and complementary bases. Upon destacking of the DNA duplex structure by protein-induced destabilization or base flipping, 2-AP fluorescence is expected to increase (22,48–50). 2-AP containing oligonucleotides (Table 1, G–I) were purchased from Midland Certified Reagent Company (Midland, TX, USA) and annealed with complementary oligonucleotides (Table 1, J and K) at equimolar concentrations. 2-AP was incorporated in short DNA substrates (22 bp) either within homoduplex DNA (Table 1, H/J) to follow flipping of nonspecific bases, or neighboring hTDG or hOGG1 target sites (G:U^F^ in a CpG context or 8oxoG:C, Table 1, G/J and I/K, respectively) to detect substrate-specific base flipping. Due to the weak fluorescence signal of 2-AP, a DNA concentration of at least 170 nM was needed to reach sufficient fluorescence intensities as determined in concentration series prior to the experiments. To separate 2-AP fluorescence from intrinsic protein (tryptophan and tyrosine) and DNA fluorescence, we chose an excitation wavelength of *λ*_ex_ = 320 nm for our fluorescence assays. Steady-state fluorescence emission spectra (340–400 nm) were acquired with a Fluoromax-4 fluorescence spectrometer (Horiba Jobin Yvon) at excitation and emission bandwidths of 4 and 6 nm, respectively. Fluorescence measurements were carried out at room temperature (25°C) in hTDG binding buffer [0.02 M HEPES pH 7.0, 0.2 mM ethylenediaminetetraacetic acid (EDTA), 2.5 mM MgCl_2_, 0.1 M NaCl] or hOGG1 binding buffer (50 mM Tris–HCl, pH 7.5, 50 mM KCl, 1 mM EDTA, 1 mM DDT). Increasing amounts of concentrated protein were titrated to DNA substrate (170 nM) to reach final protein concentrations of 0.5, 1, 2, 3 and 5 μM in the reactions (0.5, 1 and 2 μM data not shown). The samples were rapidly mixed and incubated for at least 5 min at room temperature to ensure binding was at equilibrium. To maintain constant DNA concentrations throughout the titrations, we added the same amount of DNA substrates (170 nM) to concentrated enzymes. Reference spectra were recorded for the protein in the absence of 2-AP DNA (in the same buffer solutions) that were subtracted from the emission spectra in the presence of DNA to account for contributions to the fluorescence spectra from the protein and/or buffer. All experiments were performed in duplicates of individual titrations.

For quantification of the protein-induced 2-AP fluorescence intensity change (ΔI_2-AP_), the maxima of the 2-AP fluorescence intensity at *λ*_em_ = 373 nm were determined for DNA in the absence (I_2-AP,DNA_) and in the presence of the maximum applied protein concentration (I_2-AP,DNA+protein_ at 5 μM [protein]) for hTDG and hOGG1. The relative 2-AP fluorescence change ΔI_2-AP_ induced by base flipping or destabilization is given by:

(3) \begin{equation*} \Delta {\rm I}_{2 - {\rm AP}} = {\rm I}_{2 - {\rm AP},{\rm DNA} + {\rm protein}} - {\rm I}_{2 - {\rm AP},{\rm DNA}} \end{equation*}

Fluorescence intensities were determined as averages from two different titration experiments with SD derived from the variation between experiments. The consistently higher 2-AP fluorescence intensity changes observed for hOGG1 compared to hTDG may in part be due to the different chemical environments for the two proteins; hOGG1 contains 10 tryptophans, while hTDG only has two. Furthermore, when comparing fluorescence intensities measured for the different DNA substrates (containing G:U^F^, C:oxoG or G:C, underlined base 3′ of 2-AP, see Table 1), it should be considered that 2-AP fluorescence is affected by different sequence contexts (22). Fluorescence intensities (I_2-AP,DNA_) of the different DNA substrates in the absence of protein varied more prominently with the buffer (see above for hTDG and hOGG1 binding buffers). DNA fluorescence intensities at 373 nm were measured as (1.8 ± 0.4) and (1.4 ± 0.5) x 10^5^ CPS for G:U^F^ and nonspecific (G:C) substrates, respectively, in hTDG binding buffer and (4.5 ± 2.9) and (2.7 ± 0.5) x 10^5^ CPS for the C:oxoG and G:C substrates, respectively, in hOGG1 binding buffer.

### Statistical analysis of significances

Ratios of DNA bend angle states (% of total area under Gaussian fits) and relative fluorescence intensity changes in 2-AP fluorescence-based base flipping assays were quantitatively compared for hTDG (wt and variants) and hOGG1 with different DNA target substrates. To determine the level of significance (*P < 0.05, **P < 0.01, ***P < 0.005) of differences between these different scenarios, we used a one-tailed Student's *t*-test.

## RESULTS

### Distribution of hTDG on G:U and G:T mismatch containing DNA

**Figure 1.**
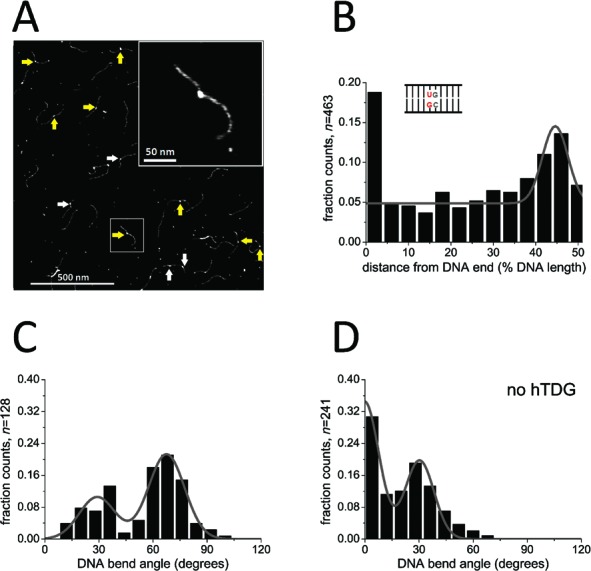
Specific complexes of hTDG at G:U lesion sites. (**A**) AFM image of the catalytically inactive hTDG variant N140A on DNA substrate containing a G:U mismatch positioned at 46% of the DNA fragment length. Arrows point to specific TDG complexes bound at the target site (yellow) and nonspecific complexes bound elsewhere on homoduplex DNA (white). The insert shows a higher magnification of a representative (specific) hTDG–DNA complex. (**B**) TDG binding position distribution on DNA demonstrating a moderate binding preference for the G:U mismatch over nonspecific DNA (enhanced occupancy at ∼46% DNA length). Fractional occupancies are plotted for ∼22 bp long sections of the 549 bp DNA substrate from DNA fragment ends (0%) to DNA center (50%). (**C**) Distribution of DNA bend angles induced by hTDG-N140A at G:U sites. A double Gaussian fit (*R*^2^ = 0.88) centered at (29 ± 10) and (68 ± 10)°. Bend angles are binned at intervals of 8 bp. (**D**) Distribution of intrinsic DNA bend angles at the G:U mismatch in the absence of protein revealed a similar DNA bend angle of (30 ± 8)° as in (C) in addition to a predominant straight DNA conformation (0 ± 8)°. Results were pooled from three individual experiments (*n* = total number of data points) and are summarized in Table 3.

For our studies, we introduced a single target site for hTDG, either a G:T or a G:U base mismatch in a CpG context, into long DNA fragments (549 bp). Figure 1A shows a representative AFM image of the catalytically inactive hTDG variant N140A incubated with DNA substrate containing a single G:U mismatch located at 46% of DNA fragment length (compare Table 1 for DNA sequence). Statistical position distributions of hTDG complexes on mismatch containing DNA (Figure 1B and Supplementary Figure S4B, black bars) can be fitted with a Gaussian curve (gray line) with maximum at ∼46% of DNA full length consistent with the position of the mismatch, indicating a preference of hTDG for target site binding over binding to nonspecific homoduplex DNA. From the Gaussian fits, we calculated moderate hTDG specificities *S* of 163 ± 47 and 305 ± 95 for the G:U and G:T mismatch in a CpG context, respectively (Table 2). Experiments carried out at higher protein concentration (2.5 μM) for the G:T containing substrate gave very comparable results (apart from slightly higher nonspecific background binding, data not shown).

**Table 2.** DNA target site specificities of hTDG

Protein	DNA substrate	AFM specificity *S*	*n*
hTDG N140A	dsDNA G:U	163 ± 47	371
hTDG N140A	dsDNA G:T	305 ± 95	364
hTDG R275A	dsDNA G:U^F^	358 ± 101	375

Binding specificities (*S*) were determined from AFM data as means ± SD from three experiments for each combination of protein variant and DNA substrate.

Due to local helix destabilization at DNA fragment ends, hTDG also displays DNA end binding preference (compare bar at 0% with ∼0.18 fraction of counts with bar at 45% with ∼0.13 fraction of counts in Figure 1B). A propensity to bind to DNA ends has also previously been observed for the glycosylase AlkA as well as many other DNA repair proteins (7,43,44,51,52).

Interestingly, volume analyses of the DNA-bound protein peaks showed that these complexes predominantly (>80%) contained only a single monomer of hTDG (Supplementary Figure S2). In contrast to some of the crystal structures picturing a weak dimer of hTDG bound to DNA (31,36,38), only a minor dimeric population was hence observed in our images, for low protein concentrations such as employed here (50 nM) as well as incubations at 2.5 μM of hTDG.

### DNA bending and base flipping by hTDG upon lesion encounter

Specific and nonspecific hTDG–DNA complexes can be distinguished based on their position on the DNA substrates (specific site at 46% of the DNA length) and were treated separately for further DNA bend angle analyses (see Materials and Methods, AFM data analysis). Our AFM data show comparable results for G:U and G:T mismatches (Figure 1 and Supplementary Figure S4, *P* = 0.1437 not significantly different, Table 3). In the following text, we will hence mainly focus on specific complexes of hTDG at G:U mismatch sites.

**Table 3.** DNA bend angles of protein–DNA complexes (hTDG, hOGG1) and intrinsic DNA bending in the absence of protein

Protein	DNA substrate	Straight [°]	Slightly bent [°]	Strongly bent [°]	*n*
Protein-induced bending					
hTDG wt	Nsp DNA (1813 bp)	NA	33 ± 11 (46%)	68 ± 11 (54%)	442
hTDG N140A	G:U	NA	29 ± 10 (33%)	68 ± 10 (67%)	128
	G:T	NA	26 ± 15 (42%)	65 ± 15 (58%)	155
	Nsp DNA (549 bp)	NA	31 ± 12 (39%)	65 ± 12 (61%)	247
hTDG R275A	Nsp DNA (1813 bp)	-2 ± 13 (57%)	34 ± 13 (36%)	65 ± 13 (7%)	424
	G:U^F^	0 ± 12 (45%)	37 ± 12 (40%)	66 ± 12 (15%)	215
hOGG1 wt	Nsp DNA (1813 bp)	0 ± 9 (44%)	33 ± 9 (20%)	70 ± 9 (36%)	450
Intrinsic DNA bending					
none	Nsp DNA (549 bp)	0 ± 18	NA	NA	209
none	Nsp DNA (1813 bp)	0 ± 23	NA	NA	260
none	Nsp DNA (549 bp and 1813 bp pooled)	0 ± 21	NA	NA	469
none	G:U mismatch	0 ± 8 (64%)	30 ± 8 (36%)	NA	241
none	G:U^F^ mismatch	-2 ± 12 (63%)	33 ± 12 (37%)	NA	198
none	G:T mismatch	0 ± 9 (68%)	31 ± 9 (32%)	NA	228
none	8oxoG:C	-2 ± 15 (78%)	36 ± 15 (22%)	NA	181

Depending on DNA bending (∼0, ∼30 and ∼70°), complexes are classed as ‘straight’, ‘slightly bent’ or ‘strongly bent’. The given values represent the maxima of single, double or triple Gaussian fits to pooled bend angle distributions. The different degree of population of the different bend angle states is given in brackets as % of total area from integration of the Gaussian peaks. The total number of data points is indicated as *n*. NA = not applicable.

Measurements of DNA bend angles for hTDG-N140A bound to G:U (lesion encounter complexes, Figure 1C and Table 3) resulted in a distribution with two distinct populations. A double Gaussian fit to the distribution yielded peaks centered at (29 ± 10) and (68 ± 10)° (*R*^2^ = 0.88). Integration of the peak areas revealed that 33% of the complexes are in the less bent state and 67% are in the stronger bent state. Corresponding experiments with G:T mismatch DNA resulted in bend angle populations of 42% with (26 ± 15)° and 58% with (65 ± 15)° bend angle (Supplementary Figure S4C). Interestingly, the less bent state (∼30°) was also clearly found at the DNA mismatch sites in the absence of protein (36 and 32% of total counts for G:U and G:T, respectively), in addition to the straight DNA conformation (semi-Gaussian peak at 0°, Figure 1D and Supplementary Figure S4D) that is typically observed at undamaged sites (43,53). This finding suggests that the G:U (and G:T) mismatch itself induces an intrinsic bend of ∼30° in the DNA backbone (under the conditions of our experiments, see below in Discussion), which also prevails in the specific, mismatch-bound complexes in equilibrium with the stronger bent state (bend angle of ∼70°).

**Figure 2.**
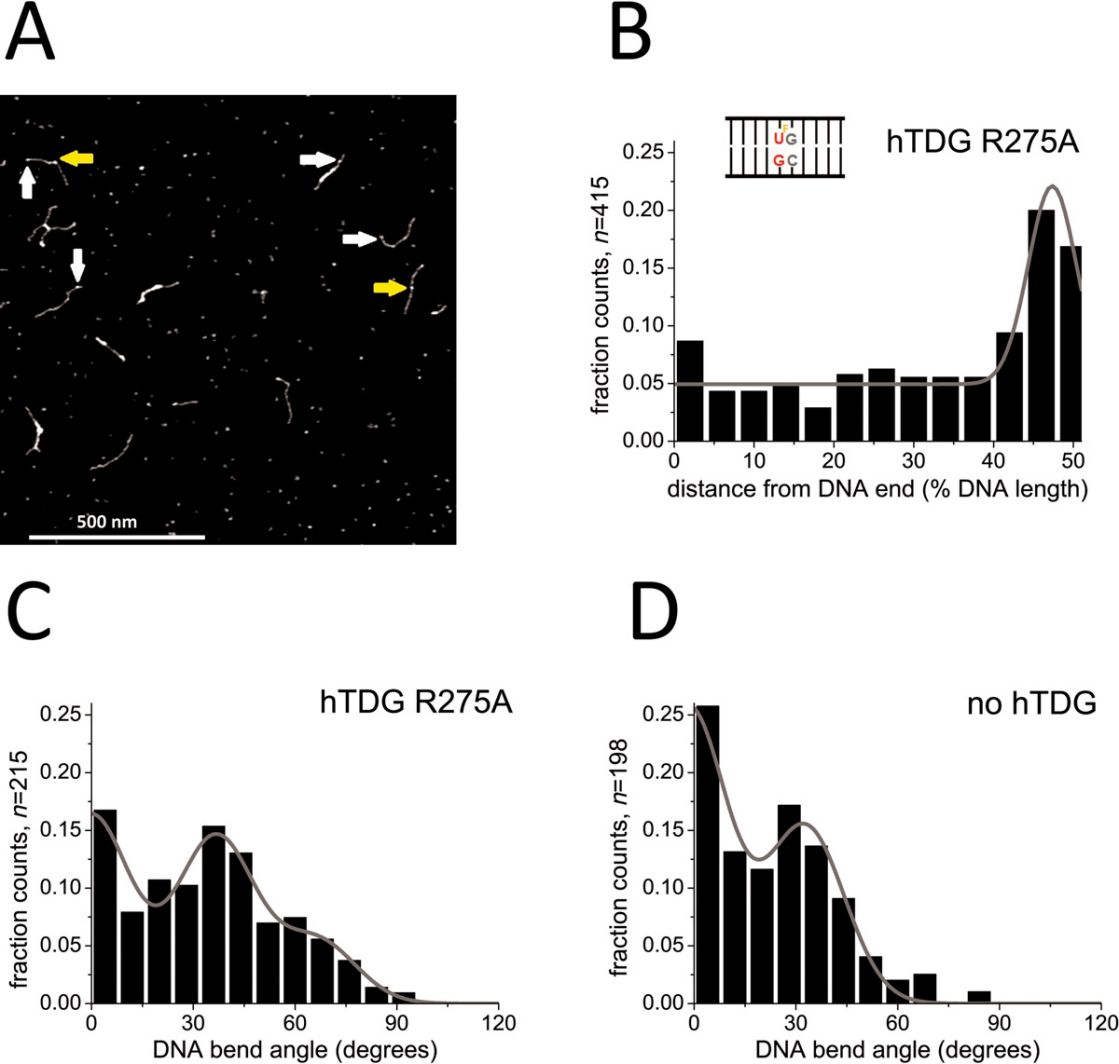
Specific complexes of hTDG-R275A at G:U^F^ lesion sites. (**A**) AFM image of hTDG-R275A on DNA substrate containing a G:U^F^ mismatch at 46% of DNA fragment length. Arrows point to specific hTDG-R275A–DNA complexes bound to the target site (yellow) and nonspecific complexes (white). (**B**) A Gaussian fit to the hTDG-R275A binding position distributions on G:U^F^ DNA revealed a lesion specificity of *S* = (358 ± 101). (**C**) Distribution of DNA bend angles induced by hTDG-R275A at G:U^F^ sites. A triple Gaussian fit (*R*^2^ = 0.89) centered at (0 ± 12), (37 ± 12) and (66 ± 12)°. (**D**) Distribution of intrinsic DNA bend angles at the G:U^F^ mismatch in the absence of protein revealed a similar slightly bent state (33 ± 12)° as in (C) and a predominant linear state (2 ± 12)°. Results were pooled from three individual experiments (*n* = total data points) and are summarized in Table 3.

We further studied DNA binding and bending by the hTDG variant R275A (Figure 2). Crystal structures of DNA-bound hTDG (to the abasic site analog THF and to the noncleavable G:U analog G:U^F^) revealed that the strictly conserved residue Arg^275^ is located at the tip of an arginine finger (36,38). Based on these structural findings and further pre-steady-state kinetics experiments, the arginine finger had been implicated in promotion and/or stabilization of nucleotide flipping in TDG by penetrating the DNA minor groove and filling the void created by base extrusion (40). Complex position analysis from our AFM images revealed a lesion specificity of hTDG-R275A for G:U^F^ comparable to (or even slightly higher than) the specificity of the catalytically inactive hTDG variant N140A for G:U (*S* = 358 ± 101 and *S* = 163 ± 47, respectively, Figures 1B and 2B and Table 2). AFM experiments in the absence of protein showed intrinsic bending of the DNA at the site of the G:U^F^ mismatch by (33 ± 12)° (Figure 2D), similar as observed for the G:U mismatch (see above and Table 3). Gaussian fits to the DNA bend angle distribution from AFM images of hTDG-R275A lesion encounter complexes bound to the G:U^F^ target site (Figure 2C, Table 3) showed an equilibrium between three states (∼0, ∼30 and ∼70°). In striking contrast to N140A, the percentage of kinked protein complexes (with a bend angle of ∼70°) is significantly reduced for R275A (67% for N140A at G:U versus 15% for R275A at G:U^F^, *P* = 0.0005, Figures 1C and 2C and Table 3).

**Figure 3.**
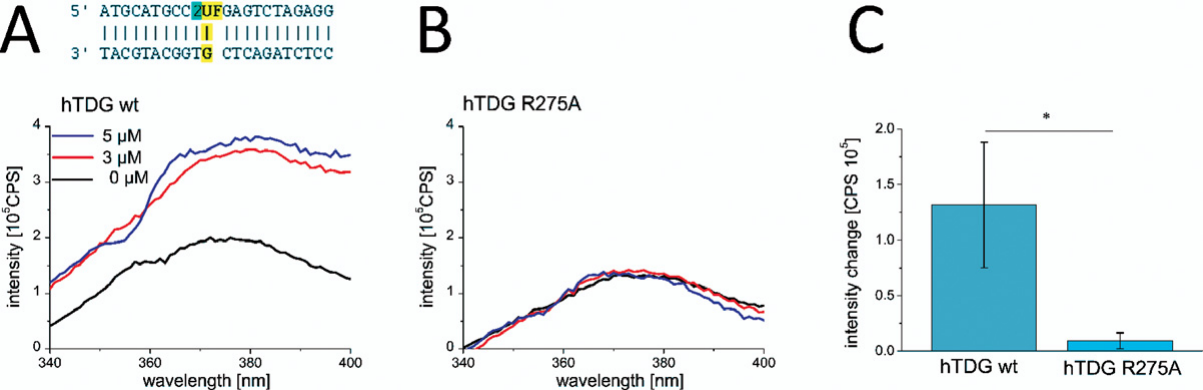
Base flipping activities of TDG. Increasing concentrations of (**A**) hTDG wt and (**B**) hTDG-R275A were titrated to 170 nM 2-AP DNA substrates. Steady-state fluorescence emission spectra (340–400 nm) were recorded at *λ*_ex_ = 320 nm. The inset in (A) shows a schematic of the 2-AP (2, cyan) DNA substrate containing G:U^F^ (yellow). (**C**) Quantification of 2-AP fluorescence intensity increases in (A) and (B) in the presence of 5 μM protein. Results were derived from two individual titrations. The error bars indicate the SD. Significance is classed as *P < 0.05.

Furthermore, we placed 2-AP as a fluorescent probe next to G:U^F^ to investigate specific DNA target site interactions of hTDG and monitor base flipping (Figure 3). As described in previous studies, the fluorescence of this adenine analog can be used to monitor unstacking of a neighboring base from the DNA, in our case unstacking of the mismatched uracil (22,49,50). In titrations with hTDG wt, the 2-AP fluorescence intensity increases with increasing [TDG] (Figure 3A), consistent with the expectation that uracil flips out of the duplex and into the TDG active site. At the highest TDG concentration examined, we measured a significant increase in 2-AP fluorescence compared to the free DNA (*P* = 0.032, left bar in Figure 3C, see Methods and Supplementary Table S1). In contrast, binding of R275A-TDG to G:U^F^ DNA does not result in a substantial increase in 2-AP fluorescence (significantly lower fluorescence than for the wt protein: *P* = 0.0466, Figure 3B and 3C, and Supplementary Table S1), suggesting a lower degree of uracil flipping for the R275A variant. This result is in accordance with the decreased population of strongly bent R275A complexes observed in our AFM data.

### Nonspecific complexes of hTDG on homoduplex DNA

**Figure 4.**
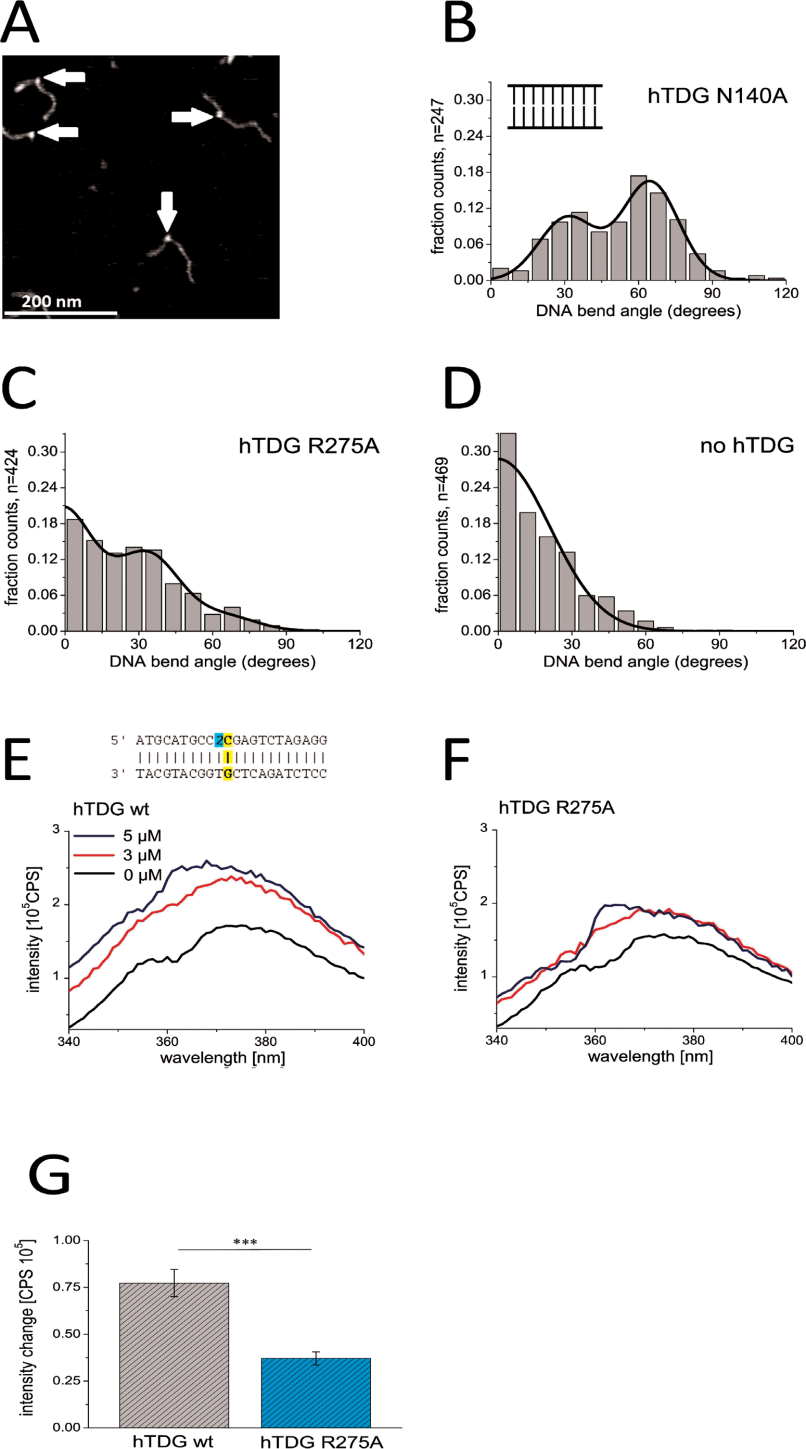
Characterization of TDG complexes with nonspecific DNA. (**A**) Representative AFM image of hTDG-N140A on nonspecific DNA (containing no lesion sites). (**B**) Distribution of DNA bend angles for hTDG-N140A complexes bound to nonspecific DNA. A double Gaussian fit (*R*^2^ = 0.94) centered at (31 ± 12), (65 ± 12)°. (**C**) Distribution of DNA bend angles of hTDG-R275A bound to nonspecific DNA. A triple Gaussian fit centered at (-2 ± 13), (34 ± 13) and (65 ± 13)°. (**D**) Intrinsic DNA bend angles of nonspecific DNA substrates. A semi-Gaussian fit centered at (0 ± 21)°. Bend angle results were pooled from at least three individual experiments (*n* = total number of data points). (**E** and **F**) Increasing concentrations of (E) hTDG wt and (F) hTDG-R275A were titrated to 170 nM 2-AP DNA substrates. Steady-state fluorescence emission spectra (340–400 nm) were recorded at *λ*_ex_ = 320 nm. The inset in (E) shows a schematic of the DNA substrate used containing a 2-AP (2, cyan) neighboring a Watson–Crick G:C base pair (yellow). (**G**) Quantification of the 2-AP fluorescence intensity increase in (E) and (F) for protein concentrations of 5 μM. Results were derived from two individual titrations. The error bars indicate the SD. Significance is classed as ***P < 0.005.

After characterizing specific TDG complexes bound at target sites of the glycosylase, we incubated hTDG with undamaged DNA fragments to analyze nonspecific complexes of the DNA repair protein during lesion search (Figure 4). Remarkably, the DNA bend angle distributions of these nonspecific complexes of hTDG-N140A (Figure 4B) revealed the same bend angle states as observed for hTDG-N140A bound specifically at a mismatch with bend angle populations centered at ∼30 and ∼70° (Figure 1C, Supplementary Figure S4C, and Table 3). DNA bend angle measurements for hTDG-R275A on nonspecific (nondamaged) DNA displayed a triphasic distribution as described above for specific, lesion-bound R275A (Figure 2C), with a predominant peak at ∼0°, a large population at ∼30° and an almost negligible population at ∼70° (Figure 4B, Table 3). Interestingly, the percentage of complexes with an average bend angle of ∼30° was similar for nonspecific complexes as for lesion encounter complexes bound at a G:U^(F)^ mismatch, for the N140A as well as the R275A hTDG variant (39 and 36% at nonspecific sites versus 33 and 40% at G:U^(F)^, Table 3), although the nondamaged DNA itself showed no intrinsic bending in control experiments in the absence of protein (Figure 4D).

Results from 2-AP base flipping assays revealed a significant fluorescence increase for nonspecific 2-AP containing DNA in the presence of increasing concentrations of hTDG wt (*P* = 0.0002 for 5 μM [hTDG], Figure 4E and Supplementary Table S1). In contrast, and consistent with our AFM results, incubation with hTDG-R275A (Figure 4F) induced no significant increase in 2-AP fluorescence (*P* = 0.356). hTDG-R275A showed significantly reduced activity compared to the wt protein (*P* = 0.0029, compare gray and blue bars in Figure 4G, Supplementary Table S1).

## DISCUSSION

### hTDG recognizes G:U and G:T target sites with moderate specificity

We addressed the key question of how DNA glycosylases such as hTDG go about finding their target sites (a G:U or G:T base mispair in a CpG context) in the huge excess of undamaged bases. Notably, the long DNA fragments used for our AFM experiments represent better mimics of the naturally occurring DNA substrates than the short oligonucleotides typically used in biochemical ensemble methods (42,43). However, in addition to the specific target site located within hundreds of nonspecific bases, these DNA substrates also contain several additional CpG sites (14% of total bases) spread over the entire DNA sequence. hTDG has been reported to bind to CpG sites in DNA with only 4-fold lower affinity and to random homoduplex DNA sequences with only 16-fold reduced affinity compared to its G:T target sites (39). The moderate degree of specificity of TDG for its target sites observed from our AFM experiments (*S* ∼ 150–300, see Table 2) is hence likely due to nonspecific background binding at the applied protein concentration (50 nM) and the 20-fold excess of protein over specific binding sites in our experiments. Importantly, our AFM data show a distinct preference of TDG for its target lesions (G:T, G:U and its analog G:U^F^) over an excess of undamaged DNA.

### hTDG exploits intrinsic flexibility at base mismatches in lesion search and recognition

**Figure 5.**
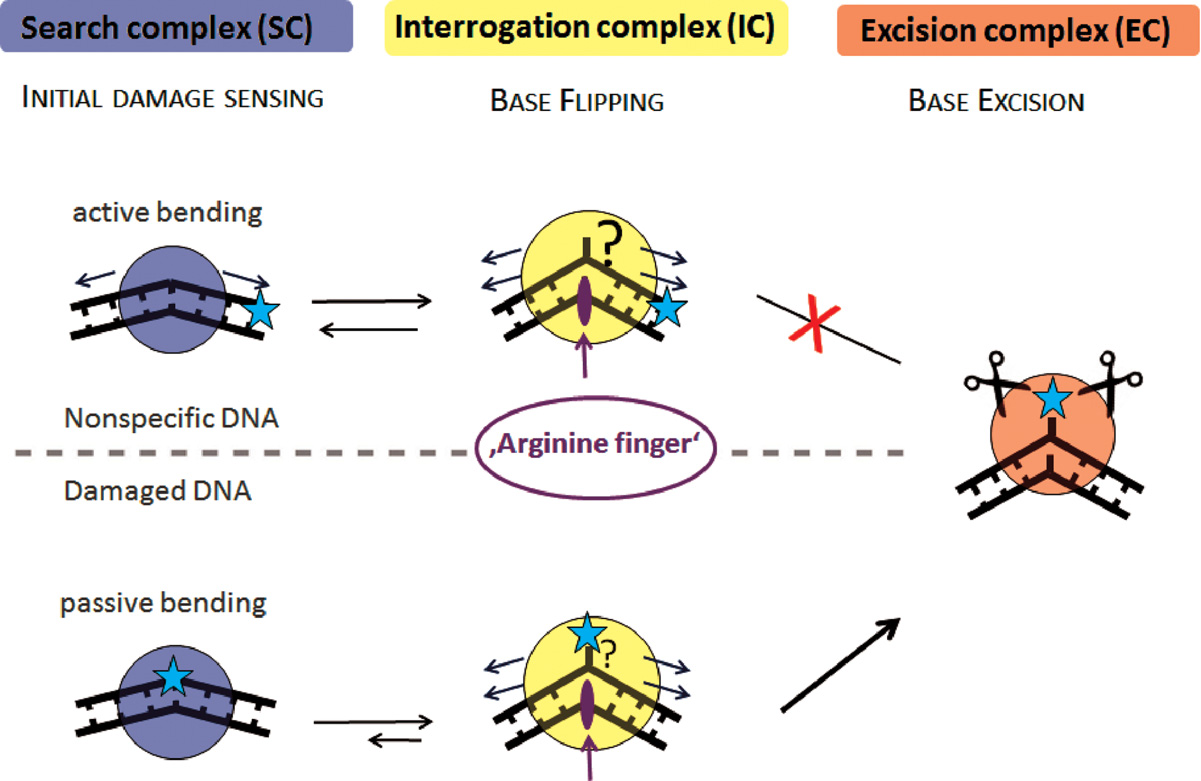
hTDG damage search model. Our proposed model for DNA lesion search and recognition by glycosylases involves three different types of TDG–DNA complexes: search complex (SC), interrogation complex (IC) and excision complex (EC). In the model, the glycosylase scans the DNA for intrinsically flexible sites, switching between an SC and an IC state. While homoduplex DNA is actively bent in the conformation of the SC (arrows indicate applied force), TDG target sites G:T and G:U form wobble base pairs (blue star) that display enhanced flexibility or intrinsic pre-bending that matches the conformation in the SC complex (passive bending). The energetic cost for DNA bending may hence serve as an initial damage-sensing mechanism that may result in a longer residence time of the glycosylase at a potential target site. In the IC, the enzyme probes base pairs via exertion of additional force on the DNA (e.g. phosphate pinching, arrows), resulting in a more strongly bent DNA structure. Protein–DNA interactions in the IC conformation may expedite spontaneous base flipping (question marks in figure) and stabilize the extrahelical base (for TDG e.g. Arg^275^). Correct target bases (or mismatched pairs) are then identified in the catalytic pocket of the glycosylase in the EC conformation and catalysis of base excision can occur.

To characterize TDG target sites in DNA (G:T, G:U, and its analog G:U^F^), we used AFM-based DNA bend angle analyses. In the absence of protein, the DNA was intrinsically bent by ∼30° at these base mismatch sites (Figures 1D and 2D and Supplementary Figure S4D). It is worth noting that in our AFM experiments, intrinsic bending at different, specific DNA sites may be somewhat promoted by the surface deposition process. However, our data still reflect the degree of destabilization at these mismatches and clearly demonstrate a propensity of the DNA to bend at these lesion sites, because bending is not observed for nonspecific DNA [nor for other lesion types tested (43)]. These findings are also in accordance with computational simulations, which show that G:T and G:U wobble pairs are kinetically less stable than canonical base pairs and are more prone to spontaneous base pair breathing (15,17,18,54,55). Glycosylases may hence have adapted to exploit these open (flipped) base configurations, stabilizing them to achieve a specific conformation required for lesion detection and removal. We will refer to this scenario as passive bending (Figure 5). Due to its higher flexibility, damaged DNA is also more prone to protein-induced DNA distortion or bending (active bending, see Figure 5), which has been shown to reduce the energetic barrier for enzymatic base flipping (13,56).

The same slightly bent state observed at G:U and G:T DNA mismatches in the absence of protein (bend angle of ∼30°) is also observed in the protein complexes bound at the mismatch sites (Figure 1C and Supplementary Figure S4C). Our data hence suggest that this bend angle state represents a complex of TDG adapted to the naturally pre-bent (or more flexible) DNA structure. Interestingly, a high fraction of these slightly bent hTDG complexes (with a bend angle of ∼30°) is also present during damage search on nonspecific DNA (Figure 4B), which (in contrast to the mismatch sites) shows no intrinsic DNA bending (Figure 4D). hTDG binding may hence impose this degree of DNA bending on undamaged DNA as part of an initial lesion-sensing mechanism. We will refer to this conformation (bend angle of ∼30°) as the lesion search complex (SC, blue circles in model Figure 5) prior to damage recognition. Bending of nonspecific DNA would induce strain on the nucleotides (active bending, Figure 5). In contrast, at its target sites, hTDG would simply stabilize the (‘pre-bent’) structure of the mispairs, which would require little or no energy for bending (passive bending, Figure 5). The energetic cost of DNA bending may serve as an initial target site-sensing mechanism by hTDG. Our findings hence suggest the search conformation to be dictated by the intrinsic structure of the target substrate.

The degree of bending observed for the SC of hTDG in our AFM experiments correlates well with the degree of DNA bending measured from crystal structures. The structures of hTDG bound to an abasic site analog (product complex) (38) and to G:U^F^ (lesion recognition complex) (36) contain a second subunit of hTDG that is bound to undamaged DNA and interacts with the specific complex via a weak dimer interface. Notably, in these nonspecific complexes, the DNA phosphate backbone is not pinched by the glycosylase and the nucleotide is not flipped into the active site (36,38). The DNA bound by the nonspecific complex is bent by ∼25° in the structures, consistent with the degree of bending observed in our AFM studies and with the idea that the bending exerted on nonspecific DNA substrates is dictated by a preformed DNA binding surface of hTDG.

The strictly conserved residues Arg^275^ and Asn^140^ of hTDG have been previously proposed to be involved in nucleotide flipping and base excision, respectively (40). In our experiments, both hTDG variants N140A and R275A are able to recognize a G:U^(F)^ or G:T mismatch similarly well as the wt protein (Figures 1B and 2B, Supplementary Figure S4B and Table 2) indicating that neither Asn^140^ nor Arg^275^ is absolutely required for initial damage recognition by hTDG. During lesion search on nonspecific DNA, the population of the slightly bent DNA state (∼30° bend angle) in both variants is also completely comparable to that in the wt protein. Our data hence suggest a model (Figure 5), which includes an initial damage-sensing step for TDG prior to base flipping and excision (represented by the slightly bent TDG–DNA structures), based on recognition of intrinsic structural properties of the target sites.

### hTDG continuously interrogates DNA during lesion search

Final target identification by hTDG as well as excision requires extrahelical base flipping. Crystal structures of hTDG (catalytic domain) bound to different target sites show the interrogated base flipped into the protein's active site pocket and the DNA strongly kinked with a bend angle of ∼45° (31,36–38). The damaged base is stabilized by active site residues, leading to an enhanced residence time of the correct (target) DNA substrate in the catalytic pocket, which results in a higher probability for base excision.

In our AFM analyses, we observed a strongly bent DNA population (with a DNA bend angle of ∼70°) in addition to the slightly bent one (∼30°) discussed above (Figures 1C and 4B and Supplementary Figure S4C). The strongly bent DNA conformation is induced by hTDG because it was not displayed by our negative controls of DNA (with or without a specific mismatch site) in the absence of protein. We interpret this strongly bent conformation as due to ‘phosphate pinching’ as seen in the hTDG–DNA co-crystal structures (31,36–38) and will refer to it as the lesion interrogation complex (IC, yellow circles Figure 5). Significantly reduced population of the ∼70° state in AFM experiments (Figures 2C and 4C) and reduced base destabilization activity in fluorescence assays for the R275A variant of hTDG (Figures 3B, C and 4F, G) support this interpretation. These findings are also consistent with the proposed essential role of Arg^275^ in base flipping and stabilization of the ‘phosphate pinch’ from crystal structures (38,40). It is worth noting that the DNA bending phenotype of hTDG-R275A in the AFM studies and its lack of base flipping activity suggested by the 2-AP fluorescence assays is not due to impaired DNA substrate binding activity of the Arg variant, because DNA binding affinities were found to be only slightly (≤ 3-fold) reduced for hTDG-R275A versus hTDG wt (39,40) (and unpublished data).

DNA bending conceivably leads to a destabilization of the interrogated base pair, consistent with an increase in 2-AP fluorescence in our base flipping assays. Base destabilization by DNA bending in the IC may serve to lower the energy barrier for spontaneous base flipping, which is then stabilized by protein–DNA interactions. Interestingly, the relative population of the strongly bent complex conformation in our experiments was slightly lower for hTDG bound to G:T versus G:U mismatch sites (58% for G:T, 67% for G:U), consistent with previous findings that flipping of T is hindered relative to U flipping (36). While the IC populations were similar (< 1.2-fold different) for nonspecific and specific complexes in our AFM studies, the base flipping assays showed an almost 2-fold enhanced fluorescence intensity for G:U^F^ versus nonspecific DNA. This small discrepancy may be an indication of (spontaneous) thermal base flipping being specifically enhanced in lesion-bound ICs by the protein-induced DNA bending. Although further studies are clearly required for a more detailed interpretation, crystal structures also showed that the formation of the specific protein contacts to the guanine base opposite the target mismatch, which may serve to further stabilize only the correct target lesion in the catalytic pocket, requires prior base flipping (38). Base flipping (whether actively induced by the enzyme or brought about in a semi- or fully-passive manner) is hence a direct precursor step toward the conformations observed for the lesion recognition complex in crystal structures.

The bend angle observed in the crystal structures at a lesion site (∼45°) is clearly smaller than the ∼70° bend angle that we measure from AFM analyses. However, in contrast to our studies, all available crystal structures of hTDG–DNA complexes are not of the full-length protein, but of a shortened catalytic domain construct that lacks the N- and C-terminal regions of hTDG (residues 1–110 and 309–410) (31,36–38). The N-terminal domain enhances binding and repair of G:T sites by hTDG (57,58). Since base flipping into the enzyme's active site is destabilized for T compared to U (36), formation of the catalytically active complex with a G:T mismatch may profit from additional support and stabilization by DNA interactions from residues in the N-terminal protein domain. Interestingly, comparison of the bending seen in our studies and in the crystal structures of the isolated catalytic core domain indicates that the N-terminal domain of hTDG may not be necessary for nonspecific binding during lesion search (comparable bending of nonspecific SC), but contribute significantly to the conformation of the specific IC complex with a target site, likely for stabilization.

Importantly, in our AFM studies, the strongly bent state (∼70° bend angle) was clearly present and similarly populated in nonspecific complexes of TDG with homoduplex DNA as in the specific TDG–lesion complexes. Significant increase in 2-AP fluorescence in our base flipping assays further supports destabilization of nonspecific DNA bases by TDG during lesion search (Figure 4E and G). The fact that both SC and IC are present both at nonspecific and at specific DNA sites strongly suggests a constant DNA interrogation process by TDG as the protein scans the DNA in search of a target site. Continuous switching between an SC and IC conformation is reminiscent of the equilibrium between an open and a closed state of DNA-bound UDG reported by J. Stivers laboratory (24) (resembling a ‘phosphate pinching’ motion) and consistent with the frequent pausing of other glycosylases observed in dynamic single molecule studies by S. Wallace's laboratory (28).

### Comparison of different glycosylases reveals general lesion search approaches

The idea that DNA repair proteins detect enhanced DNA flexibility caused by weakened base stacking at potential DNA lesions to reduce the task of finding their target sites by several orders of magnitude is highly appealing and has been put forward repeatedly (59–61). Our data provide support for this attractive theory, showing that TDG binding stabilizes DNA structural features that pre-exist at target sites. In the TDG complexes with nonspecific DNA (during lesion search), this ‘pre-bent’ DNA structure with a minor bend angle of ∼30° (which we interpret as the SC state of the glycosylase) is present in equilibrium with a more strongly kinked (∼70° bend angle) DNA state (which we interpret as the IC complex with the interrogated base destabilized and/or flipped out of the DNA double helix). To test the generality of our initial damage detection and continuous base interrogation model (Figure 5), we compare our results for TDG with those obtained on other glycosylases. In particular, we have performed identical studies on the well characterized human 8-oxoguanine DNA glycosylase (hOGG1) as a reference (Supplementary Figure S5). The two proteins hTDG and hOGG1 detect base lesions with significantly different base pairing energies. hTDG targets intrinsically flexible G:U and G:T wobble pairs that result in slightly bent structures in our AFM images (Figure 1D and Supplementary Figure S4D). In contrast, hOGG1 excises 8-oxoguanine (oxoG), which forms stable Watson–Crick like base pairs with cytosine (18,62) that lack intrinsic DNA bending and show straight conformations in AFM images in the absence of protein (Supplementary Figure S5).

In the presence of hOGG1, AFM experiments on nonspecific DNA showed an equilibrium between a straight DNA conformation (SC, matching the bend angle state of the unbound target site) and a kinked complex with bend angle of ∼70° (IC) (Supplementary Figure S5), as also previously observed (21). These results are in line with the requirement for DNA flexibility (resulting in the kinked complex) for DNA repair activity by hOGG1 observed from high speed AFM experiments (63). The same DNA bend angle of ∼70° was also observed in co-crystallographic studies of hOGG1 bound to its flipped target site 8oxoG:C (64) or bound to a nonspecific base with the base flipped into an exosite in close proximity to the enzyme's active site pocket (65). Our 2-AP base flipping assays also suggested considerable stabilization of the flipped out base by hOGG1 both at an oxoG target site and at nonspecific homoduplex sites in the DNA substrates from large fluorescence intensity increases (Supplementary Figure S5). Importantly, in our AFM studies (Figures 1 and 4, and Supplementary Figure S5), the bend angle conformations of the ICs of hTDG and hOGG1 closely resemble each other (DNA bend angle of ∼70° for both proteins), consistent with a comparable degree of DNA bending in a number of crystal structures of different DNA glycosylases bound specifically to their flipped targets (51,64,66–69). In contrast, the degree of DNA bending in the SC, which is difficult to capture by crystallographic studies due to its transient nature, differs between the two proteins: SCs of hOGG1 are predominantly straight, while those of hTDG are slightly bent. This difference between the two glycosylases in DNA bending in the SC is consistent with the innate conformations observed for their unbound target sites (Figure 1 and Supplementary Figures S4 and S5).

In our model (Figure 5), three distinct steps control correct target site identification by DNA glycosylases and minimize futile base investigation: initial damage sensing in the SC, base flipping in the IC and target identification in the protein's active site pocket in the excision complex (EC). The correlation between SC and target site conformation observed for TDG and hOGG1 in our studies may reflect an adaptation of DNA glycosylase structures to the native DNA structure and flexibility at their target lesions. Such sculpting based on structural and mechanical features of the target site presents an exciting theory for a common initial target recognition strategy of DNA glycosylases, but clearly requires further investigations. Initial damage sensing (based on the SC conformation) would likely pause DNA scanning at a potential target site, consistent with our AFM position distributions for hTDG and the preferential binding of MutM to A:T sites (rather than G:C) that are energetically closer to the target site (oxoG) of the enzyme (70). Diffusion constants measured for individual DNA glycosylase molecules from single molecule data with nonspecific DNA proved much too large to allow for sufficient time at a target site during scanning for the enzyme to capture a spontaneously flipped base (in a passive base flipping mechanism) (6). In line with this, enhanced pausing has been directly observed in single molecule DNA tightrope experiments for different DNA glycosylases in the presence of target sites in the DNA (28). Pausing may hence serve to enhance the time spent by the enzyme at a potential target site and thus to increase the probability for the residence time of the protein to coincide with (multiple events of) spontaneous, thermally induced base flipping. A passive(-uni) base flipping mechanism, in which the enzyme traps a base that is spontaneously emerging from dsDNA while the enzyme is already bound at the site has previously been put forward by J. Stivers and colleagues for the glycosylase UNG (25), which is structurally related to TDG. Our model suggests a semi-passive mechanism, in which pausing at potential target sites allows for the sampling of base flipping, which occurs spontaneously but is also energetically enhanced as well as stabilized by protein–DNA interactions (such as phosphate pinching and DNA intercalation). Consistent with this notion, active glycosylase–DNA interactions have been demonstrated to enhance the opening equilibrium of DNA in kinetic analyses (26,27,71) and faster overall diffusion (i.e. reduced pausing) was observed for glycosylase variants lacking a wedge that intercalates into the DNA and buckles potential target base pairs (28). It is conceivable that an additional energetic contribution, for instance from protein-induced DNA bending, be fine-tuned in the different glycosylases to match the specific requirements for base flipping in their particular target base pairs and under the conditions of their particular organism. Once flipped extrahelically, the target base (as well as, in the case of TDG, the opposing base) is then stabilized by protein–DNA interactions in a catalytic EC conformation, in which a steric fit of the fully flipped base into the enzyme's active site pocket is the final step in target site identification.

## CONCLUSION

To understand the poorly characterized processes of damage search before base extrusion by BER glycosylases, we studied damage detection of TDG by single molecule AFM imaging. Our results support an equilibrium between SC (slightly bent DNA structure) and IC (strongly bent DNA) states during TDG's DNA lesion scanning process, suggesting continuous testing for target sites in lesion search. Importantly, our data suggest that hTDG may exploit the intrinsic flexibility of its target mismatch sites as an initial recognition criterion leading to paused scanning and target site interrogation. Strong DNA bending in the IC (consistent with phosphate pinching seen in crystal structures) likely reduces the stability of the interrogated base pair, increasing the probability for spontaneous base flipping in a semi-passive base flipping mechanism. Our AFM and fluorescence base flipping data indicate an important role of an arginine finger in hTDG for the stabilization of the bent (IC) conformation. The extrahelical base is then stabilized in the catalytic site by protein–DNA interactions leading to base excision. Our data add significant information to previous findings on other glycosylases toward a better understanding of the complex potentially common strategy for target search and recognition by BER glycosylases.

## SUPPLEMENTARY DATA

Supplementary Data are available at NAR Online.

SUPPLEMENTARY DATA
