# Supplementary material for: Lesion search and recognition by thymine DNA glycosylase revealed by single molecule imaging
Source: Nucleic Acids Res. 2015 Feb 24;43(5):2716–29. doi: 10.1093/nar/gkv139 (PMC4357730; doi:10.1093/nar/gkv139)
Supplement: SUPPLEMENTARY DATA [file supp_43_5_2716__index.html]

Lesion search and recognition by thymine DNA glycosylase revealed by single molecule imaging — SUPPLEMENTARY DATA 

# Lesion search and recognition by thymine DNA glycosylase revealed by single molecule imaging

## SUPPLEMENTARY DATA

**Files in this Data Supplement:**

- SUPPLEMENTARY DATA
