# Supplementary material for: Lesion search and recognition by thymine DNA glycosylase revealed by single molecule imaging
Source: Nucleic Acids Res. 2015 Feb 24;43(5):2716–29. doi: 10.1093/nar/gkv139 (PMC4357730; doi:10.1093/nar/gkv139)
Supplement: SUPPLEMENTARY DATA [file supp_gkv139_nar-03509-h-2014-File007.pdf]

Supporting Information to

Lesion search and recognition by thymine DNA glycosylase revealed by single molecule imaging

by Claudia N. Buechner, Atanu Maiti, Alexander C. Drohat, and Ingrid Tessmer

Suppl. Table S1: Quantification of 2-AP fluorescence based base flipping data. 2-AP fluorescence intensity changes for the indicated proteins (5 µM) and DNA substrates. Values represent means ± SD derived from at least two individual experiments. Significances are given as P-values for the differences between hTDG variant R275A versus wt protein for lesion containing and nonspecific DNA substrate, and for hOGG1 with specific 8oxoG:C DNA versus hOGG1 with nonspecific DNA substrate. Significance levels are denoted as * for $P < 0.05$ (low significance) and *** for $P < 0.005$ (highly significant).

Protein	DNA substrate	rel. intensity change	P
hTDG wt	dsDNA 2-AP G:U^F	131555 ± 56391	-
hTDG R275A	dsDNA 2-AP G:U^F	9370 ± 7085	0.0466 *
hTDG wt	dsDNA 2-AP nsp	77297 ± 7239	-
hTDG R275A	dsDNA 2-AP nsp	37045 ± 3514	0.0029 ***
hOGG1 wt	dsDNA 2-AP nsp	145780 ± 14425	-
hOGG1 wt	dsDNA 2-AP oxoG	908275 ± 208547	0.0177 *

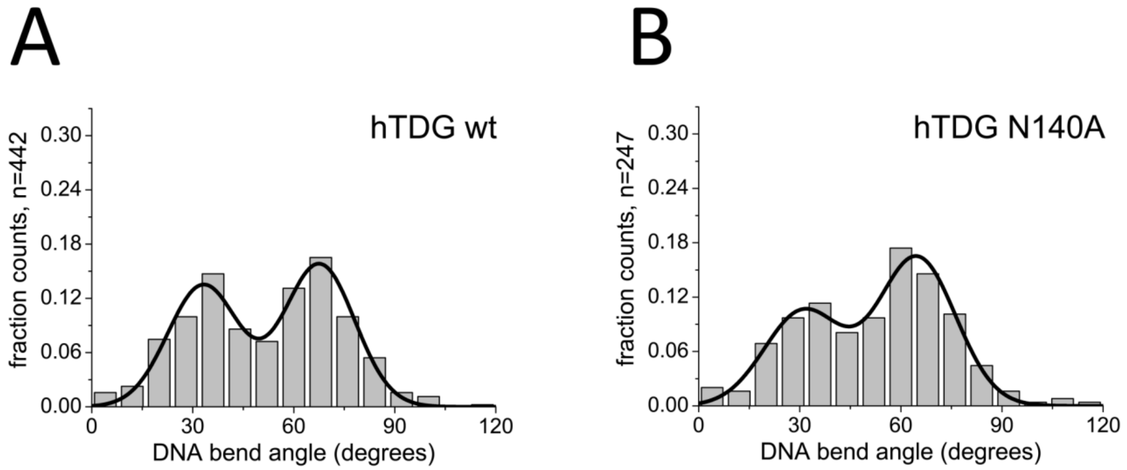

Suppl. Figure S1: Comparable DNA interactions for hTDG wt and hTDG-N140A. Distributions of DNA bend angles induced by hTDG wt (A) and hTDG-N140A (B) at nonspecific DNA sites. A double Gaussian fit (R^2 = 0.96) centered at (33 ± 11)° (46 %) and (68 ± 11)° (54 %) for the wt protein. Bend angles for hTDG-N140A on nonspecific DNA were (31 ± 12)° (39%) and (65 ± 12)° (61%) and (R^2 = 0.94), showing no significant difference compared to the wildtype protein (P = 0.478). The distributions were pooled from three individual experiments (n=total number data points).

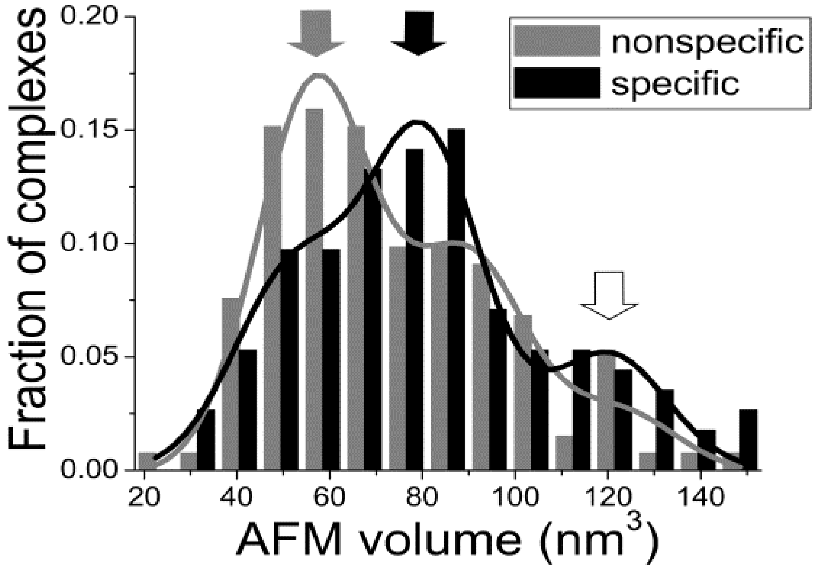

Suppl. Figure S2: AFM volume distributions of hTDG at specific and non-specific DNA sites. Volume distributions after incubation at 2.5 µM hTDG and a protein:DNA ratio of 10:1 showed three different states indicated by arrows (Gaussian fits to the distributions with $R^2 > 0.95$, n = 113 for specific and 132 for nonspecific complexes). Even at this high protein concentration, only <~20% of DNA bound complexes display the largest volume (white arrow, corresponding to ~100 kD before subtraction of the included DNA volume), which may represent a dimer or two closely bound monomers of hTDG (monomeric MW ~45 kD). The two major conformations (gray and black arrows) reveal volumes consistent with monomeric states. Different contributions from the DNA to the apparent overall topographies in the different conformational complex states (with bend angles of ~30° and ~70°) may be responsible for the two distinct volume states. Interestingly, the populations of the two states differ for complexes at non-specific sites and at the mismatch (here G:T): the equilibrium appears to be shifted towards the conformation represented by the larger monomeric volume state (black arrow) for complexes bound at a specific site (black bars). Preliminary data indicated that this state may correspond to the ~70° bend angle state.

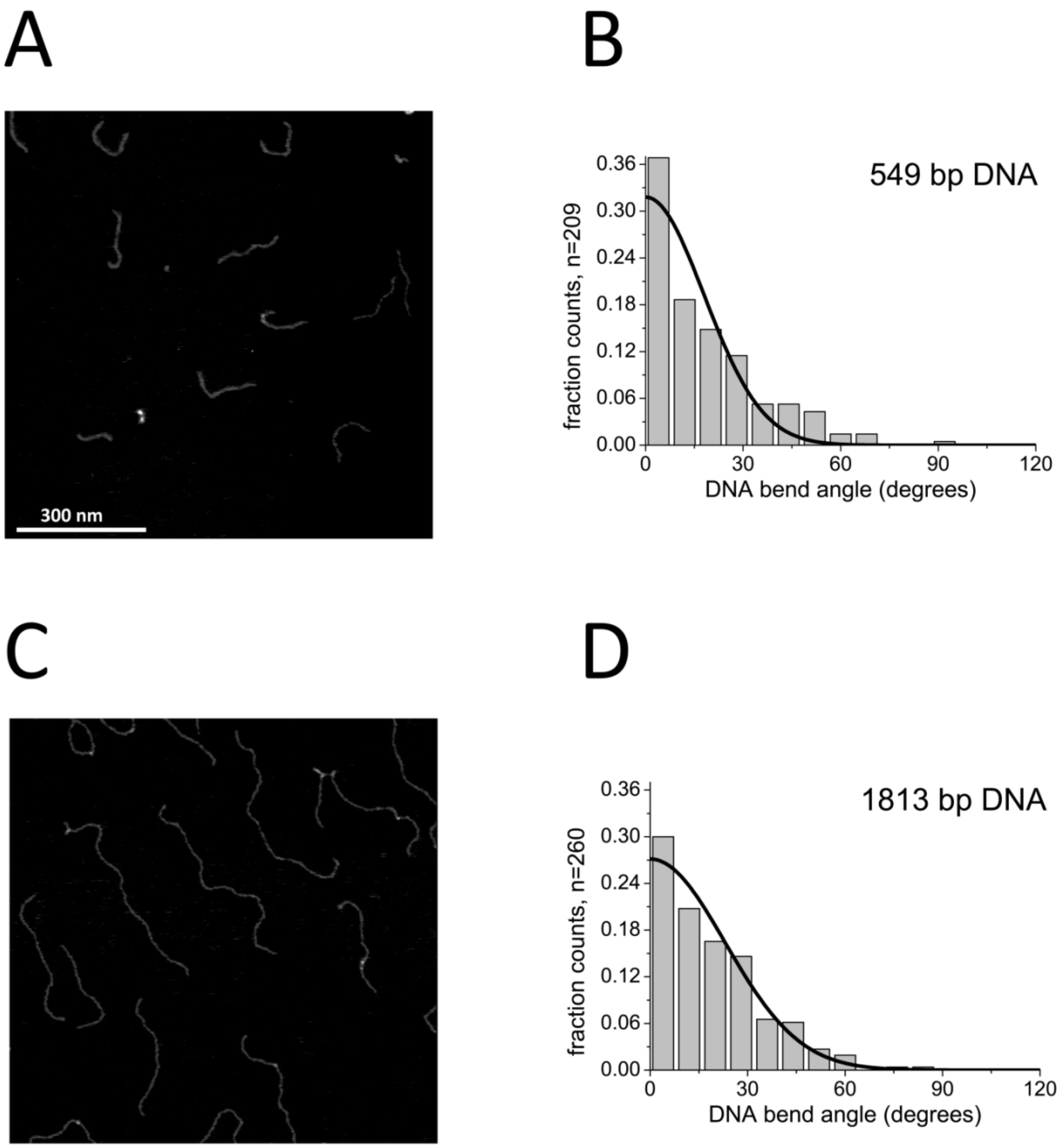

Suppl. Figure S3: Intrinsic bending of nonspecific DNA. A,B) Using the same DNA substrate as in experiments with lesion containing DNA (549 bp), but without any base mismatches or lesions (nonspecific substrate), we measured intrinsic bending at 46% of the DNA fragment length (target site position in the specific DNA substrates) to exclude any influence of DNA sequence context on intrinsic DNA bending behavior. A semi-Gaussian fit centered at (0 ± 18)° (R^2 = 0.895). C,D) Intrinsic bending within a 1813 bp DNA fragment (that did not contain any base mismatches or lesions) was determined by moving a mask comparable to the size of TDG at regular intervals of 50 nm along the DNA. A semi-Gaussian fit centered at (0 ± 23)° (R^2 = 0.963). Results were pooled from two individual experiments (n = total number data points). Both AFM images A) of the 549 bp fragments and C) of the 1813 bp fragments are 1 µm x 1 µm.

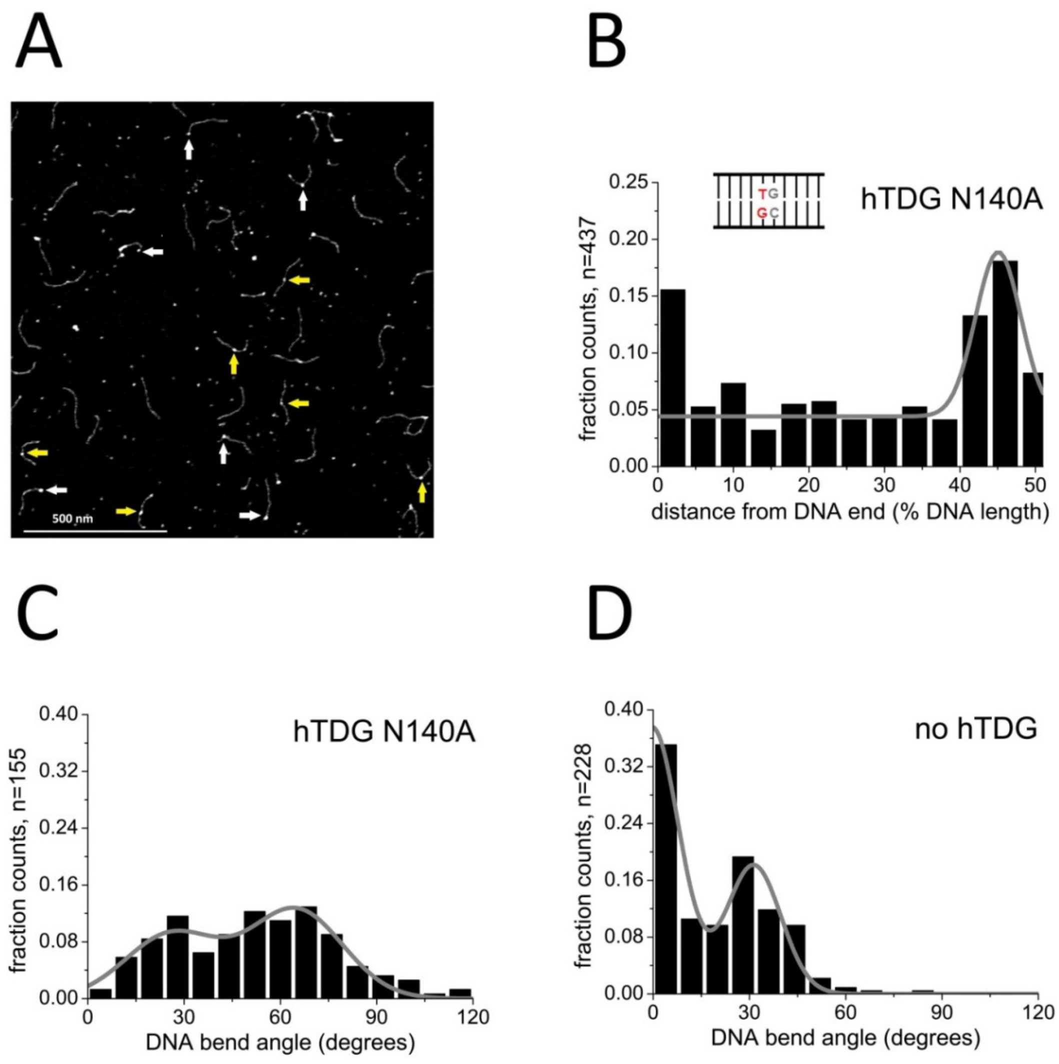

Suppl. Figure S4: Specific complexes of TDG at G:T lesion sites. A) AFM image of hTDG-N140A on DNA substrates containing a G:T mismatch at 46% of DNA fragment length. Arrows point to specific TDG complexes bound to the target site (yellow) and nonspecific complexes (bound elsewhere on homoduplex DNA, white). B) hTDG-N140A binding positions demonstrate a moderate binding preference for the G:T mismatch, with specificity S = 305 ± 95 determined from a Gaussian fit to the protein position distribution. Fractional occupancies of the different positions along the DNA are plotted for ~22 bp long segments of the 549 bp DNA substrate, from DNA fragment ends (0%) to DNA center (50%). C) Distribution of DNA bend angles induced by hTDG-N140A at G:T sites. A double Gaussian fit (R^2 = 0.87) centered at (26 ± 15)° (42%) and (65 ± 15)° (58%). D) Distribution of intrinsic DNA bend angles at the G:T mismatch in the absence of protein revealed similar DNA bending by (31 ± 9)° (32%) as in (C) in addition to a predominant straight DNA state (0 ± 9)° (68 %). Results were pooled from three individual experiments (n = total number data points).

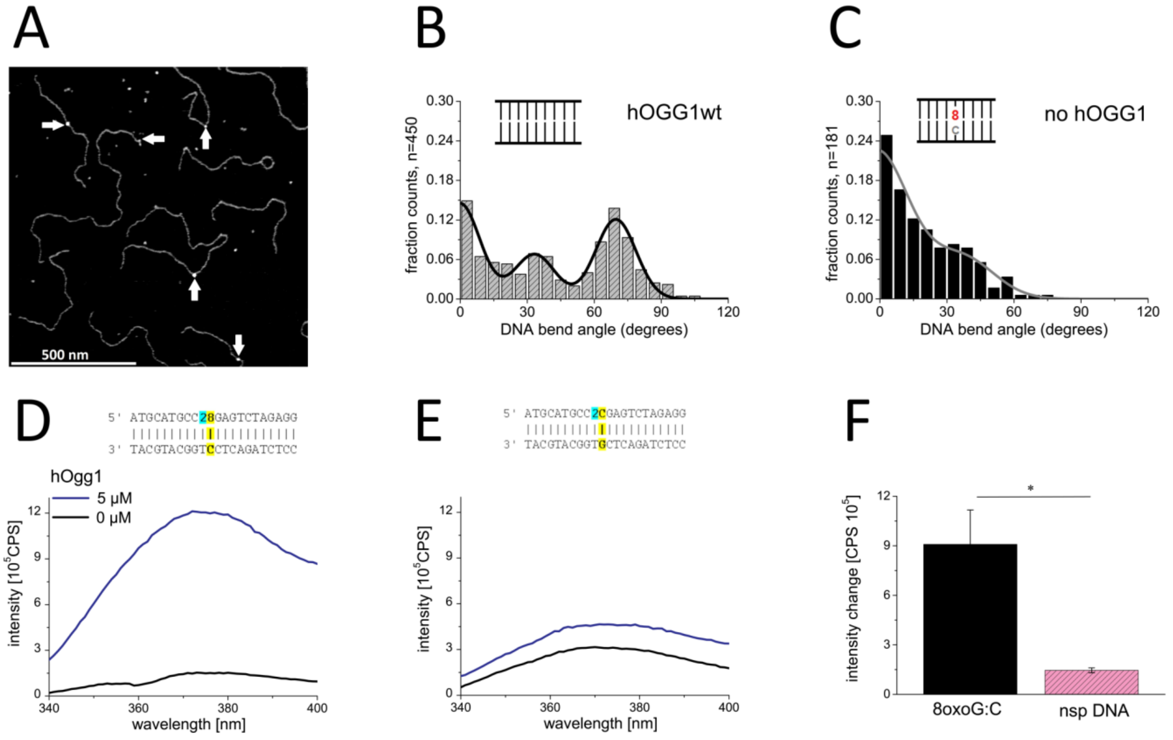

Suppl. Figure S5: Characterization of hOGG1 by AFM (A-C) and fluorescence analyses (D-F).

A) AFM image of hOGG1 binding to nsp DNA (1813 bp). Arrows point to hOGG1-DNA complexes. B) Distribution of DNA bend angles for hOGG1 bound to nonspecific DNA. A triple Gaussian fit (R^2 = 0.87) centered at ~0° (44%), ~30° (20%) and ~70° (36%). C) Intrinsic DNA bend angles at an 8oxoG site (target site of hOGG1) in the absence of protein. A double Gaussian fit (R^2 = 0.929) revealed a dominant straight fraction with bend angle ~0° (78%) and a small shoulder at ~30° (22%). Bend angle results were pooled from at least three (or two in (C) individual experiments (n = total number data points). D,E) hOGG1 was titrated to 2-AP DNA substrates containing 8oxoG:C (D) or nonspecific 2-AP DNA substrates (E). Here, only the fluorescence intensities in the absence (black curve) and in the presence of hOGG1 (blue curve at 5 µM [protein]) are shown. Steady-state fluorescence emission spectra (340 - 400 nm) were recorded at Λ_{ex} = 320 nm. The insets show schematics of the employed 2-AP (cyan) DNA substrates containing either an 8oxoG:C pair (D, yellow) or a stable Watson-Crick base pair (E, yellow). F) Quantification of the 2-AP fluorescence intensity changes in (D) and (E) for a protein concentration of 5 µM. Significances are classed as * $P < 0.05$. Results are averages from two individual titrations with error bars indicating the standard deviations. The relative 2-AP fluorescence increase for hOGG1 is consistently higher than for hTDG, most likely due to the different chemical environments of the two proteins (see Methods). In addition, the increase in 2-AP fluorescence for the specific DNA substrate (containing 8oxoG) compared

to nonspecific, homoduplex DNA (P = 0.0177) is significant, while hTDG induces a larger degree of 2-AP destabilization for nonspecific DNA relative to specific (G:U^F containing) DNA (P = 0.085).
